# Fish oil supplementation in chronic obstructive pulmonary disease: feasibility of conducting a randomised controlled trial

**DOI:** 10.1186/s40814-017-0211-2

**Published:** 2017-11-25

**Authors:** Ashley S. Fulton, Alison M. Coates, Marie T Williams, Peter R. C. Howe, Manohar L. Garg, Lisa G. Wood, Peter Frith, Alison M. Hill

**Affiliations:** 1 Alliance for Research in Exercise, Nutrition and Activity, Sansom Institute for Health Research, School of Health Sciences, University of South Australia, Adelaide, South Australia 5000 Australia; 2 Clinical Nutrition Research Centre, School of Biomedical Sciences & Pharmacy, University of Newcastle, Callaghan, New South Wales 2308 Australia; 3 Respiratory Medicine, Flinders University, Faculty of Health Sciences, Repatriation General Hospital, Adelaide, South Australia 5041 Australia; 4 School of Pharmacy and Medical Sciences, University of South Australia, Adelaide, South Australia 5000 Australia

**Keywords:** Chronic obstructive pulmonary disease, COPD, Fish oil, Omega-3 fatty acid, Feasibility

## Abstract

**Background:**

Long-chain omega-3 polyunsaturated fatty acids (LCn-3PUFAs) may act as an effective adjunct therapy for chronic obstructive pulmonary disease (COPD), a condition characterised by persistent airflow limitation and inflammation. However, the nature of this illness presents challenges for evaluating potential benefits. The aim of this study was to determine the feasibility of undertaking a randomised controlled trial of LCn-3PUFA supplementation in adults with COPD.

**Methods:**

A 16-week parallel, double-blind, randomised, placebo-controlled dietary supplementation trial was conducted. Participants diagnosed with COPD were randomly allocated to take six 1-g capsules of fish oil (3.6 g LCn-3PUFA) or corn oil (placebo) daily for 16 weeks. Key outcomes used to determine the feasibility of the trial included recruitment rate, participant retention rate and supplement adherence (blood biomarker and returned capsule count). An estimate of the effect size for clinical outcomes such as pulmonary function and functional exercise capacity was calculated.

**Results:**

None of the key feasibility criteria were met. The enrolment target was 40 participants in 52 weeks; however, only 13 were finally enrolled, with just seven in the first 52 weeks. Eight participants completed the study (retention rate 62%). Targets for compliance were not achieved; red blood cell LCn-3PUFA content (expressed as percentage of total fatty acids) did not increase by more than 2% in the fish oil group; capsule counts were unreliable. As the target sample size was not achieved and only a small number of participants completed the study, it was not possible to use the variance in clinical outcomes to estimate a sample size for a future study.

**Conclusions:**

This study highlights major difficulties, especially with recruitment, in conducting this LCn-3PUFA supplementation trial in people with COPD, rendering the protocol unfeasible by predetermined criteria. A modified approach is needed to investigate potential health benefits of fish oil in people with COPD. A multicentre study with changes to inclusion and exclusion criteria is recommended.

**Trial registration:**

Australia and New Zealand Clinical Trials Register (ANZCTR), ACTRN12612000158864

**Electronic supplementary material:**

The online version of this article (10.1186/s40814-017-0211-2) contains supplementary material, which is available to authorized users.

## Background

Chronic obstructive pulmonary disease (COPD) is a chronic lung disease characterised by persistent irreversible airflow limitation [1]. There is a significant inflammatory component in COPD, with chronic inflammation partially causing obstruction of the bronchi and leading to remodelling of the airway [1]. Long-chain omega-3 fatty acids (LCn-3PUFAs) (found predominantly in fish) have been shown to have anti-inflammatory and pro-resolutory actions in the body [2]. There are two LCn-3PUFAs that are of particular importance in inflammation: eicosapentaenoic acid (EPA) and docosahexaenoic acid (DHA), both of which are found in fish oil.

The concept that anti-inflammatory and pro-resolutory actions of LCn-3PUFAs may act as an effective adjunct therapy in people with COPD was the driver for the proposed research. However, before attempting to assess the efficacy of LCn-3PUFAs, it is important to establish the feasibility of the study protocol. At the commencement of the study, there were no similar published trials in this population and there are recognised issues that may threaten the success of an efficacy study, such as slow recruitment, intervention non-compliance and high attrition (potentially due to polypharmacy, multi-morbidity, disease morbidity, and COPD exacerbations).

Due to a lack of pre-existing data on LCn-3PUFA supplementation in people with COPD, this research aimed to determine the feasibility of undertaking a randomised controlled trial of LCn-3PUFA in people with COPD. The objectives were to (1) validate recruitment, enrolment and retention processes including exclusion/inclusion criteria and reasons for attrition; (2) validate strategies for compliance monitoring; (3) collect data on exacerbations; (4) collect data on supplement safety and (5) identify an appropriate primary outcome measure from which to calculate sample size for a larger study. As this is a randomised controlled trial, the CONSORT extension for pilot and feasibility study guidelines for reporting parallel group randomised trials were followed. The completed CONSORT checklist can be found in Additional file 1.

## Methods

A full description of methods is available elsewhere [3]. Briefly, this was a 16-week parallel, double-blind, randomised, placebo-controlled dietary supplementation trial conducted at a metropolitan hospital in Adelaide, South Australia. The trial was registered prior to commencement on the Australia and New Zealand Clinical Trials Register (ANZCTR): ACTRN12612000158864.

### Participants

Eligible participants were adults aged 18 years or over with a clinical and spirometric diagnosis of COPD (forced expiratory volume in 1 s (FEV_1_)/forced vital capacity (FVC) < 0.7) based on GOLD criteria [1], with stable (no medications commenced or change in dose) medication for 28 days prior to screening. Exclusion criteria included habitual consumption of LCn-3PUFA supplements (> 1 g/day for ≥ 3 months), unstable (change in medication in the 28 days prior to study enrolment) or very severe COPD (FEV_1_ < 30% predicted) [1], diagnosis of α-antitrypsin deficiency, current smokers, cachexia (unintentional weight loss of > 5% within 12 months [4] or a body mass index (BMI) ≤ 18.5 kg/m^2^), morbid obesity (BMI ≥ 40 kg/m^2^), unstable comorbidities or medication use, use of systemic steroids or antibiotic medication within the 28 days prior to study enrolment, respiratory conditions not related to airflow limitation, use of warfarin, participation in a comprehensive pulmonary rehabilitation program in the previous 2 years or intending to undertake this type of rehabilitation during the intervention period and a Mini-Mental State Examination score < 23.

### Recruitment

Participants were recruited via a number of different strategies including the respiratory trials database at the study site and advertisements in The Messenger Newspaper (a free weekly local South Australian Newspaper) and in a South Australian COPD support group newsletter (AIR Newsletter). Recruitment flyers were placed in three hospitals in the local area, at respiratory physiotherapy practices, exercise physiology clinics and selected general practitioner clinics in Adelaide, South Australia. Researchers also promoted the study via local radio and print media.

### Randomisation and concealment

Participants were randomly allocated by minimisation [5, 6], based on modified Medical Research Council (mMRC) scale for breathlessness score (0, 1, 2, 3 or 4), to either the fish oil group or the placebo group. A staff member external to the project determined the computer-generated randomisation schedule.

Capsules were pre-packaged into opaque containers and labelled as A or B with the investigator details and supplementation instructions. All capsules were flavoured to mask the taste and odour. Capsules were securely stored and dispensed by the clinical trials pharmacist at the study site, thus ensuring blinding of both the participants and investigators.

### Intervention

Participants were required to take six 1-g capsules orally per day for 16 weeks. The fish oil group consumed capsules containing EPAX 6000 TG/N omega-3 concentrate (EPAX, Oslo, Norway). Each capsule contained 600 mg of omega-3 fatty acids, of which 200 mg was DHA and 300 mg was EPA. The placebo group consumed capsules containing corn oil (predominantly comprised of linoleic, oleic and palmitic acids) (EPAX, Oslo, Norway), which were identical in appearance to the fish oil capsules.

### Outcomes

A number of different outcomes were included, with the feasibility outcomes being the primary focus. Feasibility outcomes included recruitment rate, retention rate, supplement adherence, refusal rate and time lost to exacerbation. Key feasibility and clinical criteria were determined a priori based on the criteria deemed most useful for the design and conduct of a larger study. Outcome measures and associated criteria are shown in Table 1. Clinical outcomes (as planned in the original protocol [3]) included supplement safety and an estimate of effect size and 95% confidence intervals for inflammatory biomarkers, pulmonary function (spirometry, impulse oscillometry (IOS), gas transfer and plethysmography), dyspnoea (Dyspnoea-12 questionnaire and visual analogue scale), functional exercise capacity (six-minute walk test) and well-being (hospital anxiety and depression scale (HADS) and chronic respiratory questionnaire (CRQ)).

**Table 1:** Summary of feasibility and clinical outcome measures and key criteria

Outcome	Outcome measure	Key criteria
Feasibility		
Recruitment rate	*n* participants enrolled in 52 weeks	40 participants enrolled after 52 weeks.
Retention rate	*n* participants completing intervention	80% of all enrolled participants to complete
Supplement adherence rate	Change in RBC LCn-3PUFA content (expressed as percentage of total fatty acids) Capsule count at study completion	≥ 2% increase in percent of LCn-3PUFA in RBC ≥ 80% of capsules consumed
Refusal rate	*n* identified volunteers who decline to participate/be randomised	
Time lost to exacerbation	*n* days, self-reported exacerbation	
Clinical (proposed in original protocol)		
Safety	Reported symptoms from supplement	
Effect size	Inflammatory biomarkers, spirometry, impulse oscillometry (IOS), gas transfer and plethysmography, dyspnoea (Dyspnoea-12 questionnaire and visual analogue scale), functional exercise capacity (six-minute walk test), and well-being (hospital anxiety and depression scale (HADS) and chronic respiratory questionnaire (CRQ))	A positive moderate effect size (≥ 0.5) for at least CRP, dyspnoea and FEF_25–75_.

*CRP* C-reactive protein, *FEF*_*25–75*_ forced expiratory flow between 25 and 75% of forced vital capacity, *LCn-3PUFA* long-chain omega-3 polyunsaturated fatty acid, *n* number, *RBC* red blood cell

### Sample size

The target sample size for the feasibility study was 40 participants, to allow sufficient precision to enable estimations for sample size for subsequent studies. The Browne’s [7] ‘rule of thumb’ suggests that a minimum of 30 participants is required to achieve sufficient precision, and this recommendation is supported by Lancaster et al. [8]. It should be noted that guidance on sample size calculations for pilot and feasibility studies was limited at the time the protocol was published in 2013. More recent research proposes other methods of determining an appropriate sample size for pilot and feasibility studies including a stepped estimate based on effect size [9], computer simulation [10] and confidence intervals [11]. There is growing recognition that sample size justification in pilot and feasibility studies is important, although to date, there is no consensus on the most appropriate method and many of the proposed methods rely on prior knowledge of variance, which may not be available.

### Data analysis

Feasibility outcomes (primary) were analysed descriptively as frequencies and rates. In the original protocol, the proposed statistical analysis (ANOVA) was planned in order to assess variance and size of differences in outcome measures between interventions as a basis for sample size estimation for a larger trial should the protocol be deemed feasible. Due to the small sample size achieved, the planned statistical analysis for the clinical outcomes was not appropriate. Effect sizes (and confidence limits) for clinical outcomes were described using Mann-Whitney *U* test *z* scores.

### Important changes to methods after trial commencement

The recruitment period was extended, and a number of recruitment strategies were introduced after publication of the protocol in 2013 [3], in an attempt to improve participation in the study. The testing order was changed so that IOS was performed before spirometry as it is acknowledged that forced spirometry manoeuvres conducted prior to IOS can impact IOS resistance and reactance values [12]. Due to the small sample size achieved, planned statistical analysis was inappropriate, and therefore, non-parametric tests were used. It also was deemed unfeasible to analyse inflammatory biomarkers.

## Results

### Participant recruitment, refusal and retention

**Fig. 1:**
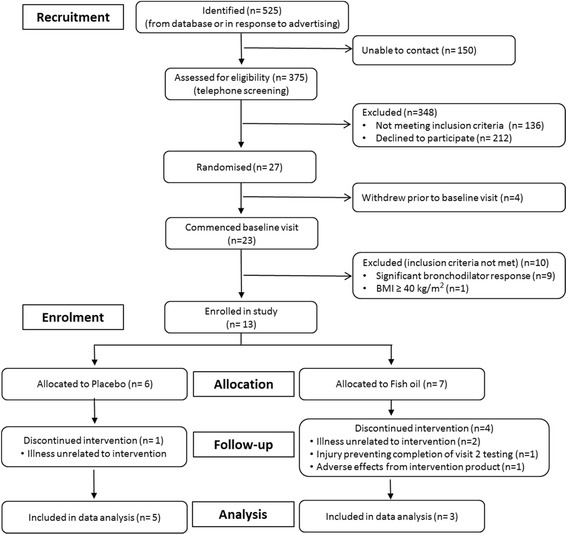
Recruitment flow diagram

Figure 1 shows an overview of participant recruitment. A total of 525 potential participants were identified between May 2013 and August 2015 from respiratory trial databases or via direct contact with researchers after seeing flyers or advertisements for the study. Researchers were unable to contact 150 potential participants from the people listed on databases, leaving 375 people who underwent a preliminary screening via telephone. Of these, 212 declined to participate and 136 were ineligible. The most common reasons people declined to participate were ‘not interested’ or ‘no reason provided’ followed by poor health and age. Of those who wanted to participate, primary reasons for ineligibility included participation in another study (*n* = 60) or fish oil consumption (*n* = 29). By far, the most effective recruitment strategy was the participant databases (503 responses), followed by advertising in The Messenger Newspaper (7 responses) and flyers on the local hospital noticeboards (5 responses).

Overall, in 2 years and 2 months, 27 participants (5% of those identified) were invited to attend the baseline visit and randomised. Of these 27 participants, only 13 participants were deemed eligible and enrolled in the study. The majority of participants were excluded at the baseline visit due to a significant bronchodilator response (*n* = 9). One participant was excluded due to a BMI > 40 kg m^2^. Within the first 52 weeks of the study, only seven participants were enrolled. Therefore, the key feasibility criterion of enrolling 40 participants in 52 weeks was not met.

Of the 13 participants enrolled in the study, eight participants completed the study (62% retention rate). One participant withdrew from the placebo group during the intervention after suffering an illness that was unlikely to be related to the intervention. Four participants withdrew from the fish oil group during the intervention. Two withdrew due to an unrelated illness (one did not attend the baseline visit), one participant suffered non-study-related injuries which prevented them from completing the test battery at visit two and one participant suffered an adverse reaction potentially related to the supplement (gastro-intestinal upset).

### Participant characteristics

Table 2 presents the baseline characteristics of the study participants (age, sex, physical characteristics, smoking status, lung function, comorbidities and medications) as median and interquartile range (IQR); only participants who successfully completed the baseline visit are included. The age of the participant refers to the age of participants on the day of the first study visit. Both the FEV_1_/FVC ratio and the FEV_1_% predicted are the post-bronchodilator measurements at the first study visit.

**Table 2:** Baseline participant characteristics

	Fish oil (*n* = 6) median (IQR)	Placebo (*n* = 6) median (IQR)
Age (years)	68.50 (2.50)	70.5 (6.11)
Sex (*n*)		
Male	3	4
Female	3	2
Height (cm)	166.80 (12.74)	167.20 (8.22)
Mass (kg)	85.00 (24.98)	82.20 (24.05)
BMI (kg/m^2^)	30.02 (6.07)	31.84 (6.07)
Smoking status (*n*)		
Never	0	1
Ex-smoker	6	5
mMRC score	1.50 (1.00)	1.00 (1.50)
Dyspnoea-12 (at rest)	12 (16)	8 (1.5)
Borg (dyspnoea, at rest)	0.5 (0.25)	0.25 (0.5)
FEV_1_/FVC	0.59 (0.05)	0.46 (0.02)
FEV_1_% predicted	72.00 (13.00)	50.00 (18.00)
Number of medications, total	6 (2)	5 (5)
Number COPD medications	2 (1)	3 (1)
Self-reported comorbidities	4 (2)	3 (2)
Charlson comorbidity score^a^	3 (1)	4 (2)
RX-Risk-V	11 (5)	14 (12)

^a^Age adjusted

*BMI* body mass index, *COPD* chronic obstructive pulmonary disease, *FEV*_*1*_ */FVC* forced expiratory volume in 1 s/forced vital capacity, *FEV*_*1*_ *% predicted* forced expiratory volume in 1 s percent predicted, *IQR* interquartile range, *mMRC* modified Medical Research Council, *RX-Risk-V* validated prescription medication-based comorbidity index

At baseline, there were few apparent differences between groups. Notably, the placebo group appeared to have more severe airflow obstruction (FEV_1_/FVC and FEV_1_% predicted) than the fish oil group. The most commonly reported comorbid conditions included hypertension (*n* = 6), cardiovascular disease (*n* = 3), diabetes (*n* = 2) and cancer (*n* = 2). A number of participants did not report conditions that they reported regularly taking medications for, most commonly cardiovascular disease (*n* = 5), reflux (*n* = 3) and depression (*n* = 1).

### Supplement adherence

#### Erythrocyte fatty acid levels

Red blood cell LCn-3PUFA content (expressed as a percentage of total fatty acids) did not increase by more than 2% in the fish oil group. However, this was likely due to one participant with an unexpectedly high LCn-3PUFA intake at baseline. The placebo group remained at similar levels or decreased slightly. In one blood sample, the fatty acids were oxidised (determined as the % of total fatty acids falling outside the normal range for key fatty acids such as 18:1n9 and 20:4n6), and therefore, results were excluded.

#### Returned capsule count

Despite several reminders to return the containers with the capsules to research staff or to the pharmacy at the completion of the trial, only one participant returned the container and there were no remaining capsules. This meant that it was not possible to determine one of the key feasibility criteria ‘≥ 80% of capsules consumed’.

### Time lost to exacerbation

No participant reported an exacerbation of COPD during the study period; therefore, it was not possible to assess the effects of exacerbations on intervention adherence in this study.

### Clinical outcomes

#### Supplement safety

Participants were asked to record in a daily diary if they experienced side effects such as burping, bloating, heartburn, stomach pain, nausea, diarrhoea, vomiting or constipation during the study. Two participants reported at least once side effect throughout the study. One participant in the placebo group reported eructation (burping) throughout the entire study, and one reported heart burn on 1 day during the intervention period in the daily diary.

#### Effect size

Only data from participants who successfully completed both visits (baseline and post intervention) were included in this descriptive analysis (detailed results for the clinical outcome measures can be found in Additional file 2). Where moderate to large effect sizes were present, the majority favoured the placebo group suggesting greater improvement or less deterioration than the fish oil group. As the study did not reach the target sample size and only a small number of participants completed the study (fish oil *n* = 3, placebo *n* = 5), it was not possible to use the variance to estimate a sample size for a future study from the results of this study.

## Discussion

The driver for the research was the efficacy of fish oil; however, this study focussed on the feasibility of the study protocol and was never intended to test efficacy. The results indicate that the study protocol was not feasible with the current protocol at a single trial site. This does not necessarily indicate that the trial is not feasible but it underlines the need for protocol changes. The most important factor affecting trial feasibility was recruitment, which was hindered by a number of factors including the inclusion and exclusion criteria. A number of people that were contacted were also already taking fish oil for other chronic conditions such as osteoarthritis and cardiovascular disease; this may be a result of the widespread media and marketing of fish oil in common comorbidities of COPD.

A key issue in reporting this feasibility study revolved around the discrepancy between the prospectively planned protocol (published 2013) and the outcome of the study. We had prospectively planned the clinical outcomes in the original protocol as a basis to explore variability and effect size differences between groups as a basis for estimating sample size for a larger well-powered RCT, should the planned protocol be deemed feasible. As it turned out, this protocol could not be deemed feasible (recruitment, sample achieved, etc.) and the planned analyses for differences for clinical outcomes between the fish oil and placebo groups were deemed inappropriate. Rather than omit all reference to the clinical outcomes (and selectively report a priori outcomes) and given the sparse literature in this area, we opted to report the effect sizes (and confidence intervals) between groups (Additional file 2). We acknowledge that with such a small sample, this data may not be representative and may reflect a very conservative view of differences between the two groups; larger samples would allow greater confidence of the real difference between groups.

### Proposed protocol changes resulting from study findings

In this study, recruitment was the key factor limiting study feasibility; a number of changes to the current protocol are proposed to increase the feasibility of the study and these are outlined below and summarised in Table 3.

1. Inclusion of multiple recruitment and data collection sites: This would not only increase the pool of available potential participants but may also allow for greater external validity by allowing for metropolitan and rural sites to be included, with towns and cities of varying population size, socioeconomic status and risk factors for COPD.
2. Inclusion of participants with COPD and asthma-COPD overlap (ACO): A key component of COPD is airflow obstruction that is not fully reversible. The accepted definition of airway obstruction reversibility is an increase in FEV_1_ (L) of > 12% and 200 ml post short acting bronchodilator [13] representing significant airflow reversibility. A number of potential participants were excluded because they attained this level of reversibility. At the trial commencement, ACO was a relatively new concept, and while understanding of this category of chronic airflow limitation has increased in recent years, there is still no formal definition [13]. Including participants with ACO, determined here as people with significant airflow limitation reversibility but still abnormal lung function post bronchodilator (FEV_1_/FVC ratio of < 0.7), would not only increase the recruitment rate but would also be more representative of the general COPD population.
3. Recruitment via general practitioner networks and professional recruitment companies: Distribution of study information to the target population (people with stable COPD excluding very severe COPD) was difficult. While the database was the most effective strategy, there were a number of issues including the following: (1) all people with respiratory diseases were included, (2) a large proportion of people with COPD on the database had severe or very severe COPD making them ineligible for this study, and (3) there were multiple studies underway at the same time with similar inclusion and exclusion criteria using the database (commonly drug trials, which participants may perceive a greater benefit from participation when compared to a dietary supplement). A proposed change to the protocol is the recruitment of general practitioners (GPs) to distribute the information to the target population, as primary care providers offer an opportunity to target people with COPD, who are likely to visit their general practitioner frequently. It is recognised that there are also a number of barriers to this kind of recruitment, especially as general practitioners are usually time poor. Bell-Syer and Moffett [14] suggest emphasising the practical implications of the research, regular contact with GPs (not just practice managers) throughout the recruitment period, minimising the burden to the GP by including a standardised referral form and only providing doctors with simple and key inclusion and exclusion criteria.
4. Screen participants for erythrocyte omega-3 index (EPA + DHA) rather than self-report PUFA supplementation: Including participants who have previously or are currently consuming fish oil to participate in the study was considered as this could have potentially increased recruitment by approximately one third in the current study. However, participants would need to delay commencement of the study to allow for a washout period of 4 months, as EPA and DHA are incorporated into cell membranes for the life of the cell in a dose-dependent manner [15]. Milte et al. [15] also showed that erythrocyte EPA + DHA levels of 8% could be achieved by long-term supplementation with approximately 1 g/day of EPA + DHA (an increase of 9% was achieved from 6 g/day of fish oil over 12 weeks of supplementation). The higher the omega-3 index is at baseline, the less likely any further supplementation would elicit a benefit. It is therefore proposed that instead of screening potential participants based on self-reported intake of fish and omega-3 supplements, participants are screened based on erythrocyte omega-3 index, excluding people with an omega-3 index of ≥ 5%. It is acknowledged that while this adds a significant time burden to researchers in the recruitment phase and may reduce rather than increase recruitment, this is important for scientific rigour and an objective measure of intake would provide greater confidence in the habitual intake of fish oil.

**Table 3:** Summary of proposed protocol changes

Number	Summary
1	Inclusion of multiple recruitment and data collection sites
2	Inclusion of participants with COPD and asthma-COPD overlap (ACO)
3	Recruitment via general practitioner networks and professional recruitment companies
4	Screen participants for erythrocyte omega-3 index rather than self-report PUFA supplementation

### Comparison with relevant findings from other published studies

To date, there are few published papers on pilot or feasibility studies in COPD and dietary supplement intervention studies. Thomashow et al. [16] (Cod-Fish protocol) investigated a similar dose of LCn-3PUFAs in 40 people with COPD, finding no difference between groups for the primary endpoint (% change in flow-mediated dilation of the brachial artery) or six-minute walk distance at the end of 6-month intervention. The Cod-Fish study reached the recruitment target of 40 participants (duration of recruitment not reported) with 33 participants available at the 6-month follow-up assessment (retention rate of 83%). Other than the Cod-Fish study, there are few studies in COPD that report feasibility or pilot study results, with a tendency to focus on feasibility of the intervention mode (e.g., tele-health [17]). One recent study by Faulkner et al. [18] reported the feasibility of COPD participant recruitment to a physical activity intervention trial, with similar findings to the current study; 215 participants were invited to participate in the study, with only 14 participants completing the post-intervention assessments. Almost no studies in omega-3 fatty acids are identified as pilot or feasibility studies, and those that do report feasibility outcomes such as supplement safety and adequacy of blinding.

Although few studies have been published in the area, there may be useful lessons to learn from studies that have met their recruitment targets. To date, Thomashow et al. [16] appears to have met its recruitment target, but a full version of this study has not yet been published (available as conference abstract only). Another study that included nutritional supplementation (leucine, vitamin D and omega-3 fatty acids) as an adjunct therapy to pulmonary rehabilitation had a greater retention of participants (8/81 withdrawn) [19]. Key points of difference include recruitment of participants across multiple sites, the intervention included multiple health components (supervised exercise training, education) rather than sole supplementation and rather than excluding people who had recently completed pulmonary rehabilitation and they made this a core component of the study. A large longitudinal, national (USA), randomised, double-blind, placebo-controlled study looking at vitamin D and/or n-3PUFA supplementation is currently underway; however, to date, only the protocol for the VITAL lung study has been published [20].

### Supplement adherence and safety

Fish oil has very few contraindications, and there were no safety concerns reported by participants that were considered to be related to the investigational product with the relatively high supplementation dose used in this study. There was only one serious adverse event reported, and it was deemed unlikely to be related to the intervention product. Very few participants reported adverse side effects from supplement consumption. One participant withdrew from the study due to an adverse gastro-intestinal response, and one participant reported increased eructation (burping) throughout the intervention period. This combined with previous research [17, 18] suggests that for the majority of people, fish oil supplementation is a safe adjuvant therapy. In this small sample, exacerbations of COPD did not appear to impact participants adhering to the intervention protocol. Exacerbation frequency generally increases with disease severity, and in this study, all but two participants (who were classified as severe) were classified as having mild or moderate COPD. Exacerbations in research may be of greater concern for more advanced stages of COPD. Unfortunately, only a cursory analysis of supplement adherence was possible due to the small number of blood samples available (*n* = 6) and the lack of supplement containers that were returned by participants at the end of the study period (*n* = 1).

### Strengths and limitations of the study

The strengths of this study include the rigorous double-blind randomised controlled design and the clear predetermined feasibility criteria. While the study was always intended to have a small sample size, the target was 40 participants, yet only 13 were recruited. This limited the ability of the study to address some of the objectives, for example, calculating a sample size for a future study. The small sample size also meant that it was not viable at this time to perform blood biomarker analysis to explore changes in inflammatory biomarkers; nevertheless, erythrocyte LCn3PUFA levels were still analysed and used as a measure of compliance.

## Conclusions

While the results of this study indicate that the current study protocol is not feasible at a single trial site, the potential health effects of fish oil in people with COPD warrants further investigation. The design of a larger trial to investigate efficacy should be based on the current study with important protocol changes. Recommended changes include multicentre recruitment, additional recruitment strategies such as general practitioner networks and professional recruitment companies and changes to inclusion and exclusion criteria including an objective measure of habitual LCn-3PUFA intake (omega-3 index) and the inclusion of participants with ACO.

## Additional files

Additional file 1: CONSORT 2010 checklist of information to include when reporting a randomised trial. (DOCX 36 kb)

Additional file 2: **Table S1.** Pulmonary function results including spirometry, lung volume, impulse oscillometry and gas transfer. **Table S2.** Results of well-being questionnaires including the hospital anxiety and depression scale and the chronic respiratory disease questionnaire. **Table S3.** Six-minute walk test and dyspnoea results. (DOCX 21 kb)

## Abbreviations

ACO: Asthma-chronic obstructive pulmonary disease overlap
BMI: Body mass index
COPD: Chronic obstructive pulmonary disease
CRP: C-reactive protein
CRQ: Chronic respiratory questionnaire
DHA: Docosahexaenoic acid
EPA: Eicosapentaenoic acid
FEF: Forced expiratory flow
FEV_1_: Forced expiratory volume in 1 s
FVC: Forced vital capacity
GOLD: Global Initiative for Chronic Obstructive Lung Disease
GP: General practitioner
HADS: Hospital anxiety and depression scale
IOS: Impulse oscillometry
IQR: Interquartile range
LCn-3PUFA: Long-chain omega-3 polyunsaturated fatty acid
mMRC: Modified Medical Research Council
RBC: Red blood cell
RX-Risk-V: Validated prescription medication-based comorbidity index
