# Supplementary material for: Fish oil supplementation in chronic obstructive pulmonary disease: feasibility of conducting a randomised controlled trial
Source: Pilot Feasibility Stud. 2017 Nov 25;3:66. doi: 10.1186/s40814-017-0211-2 (PMC5702222; doi:10.1186/s40814-017-0211-2)
Supplement: Supplementary file 1 — CONSORT 2010 checklist of information to include when reporting a randomised trial. (DOCX 36 kb) [file 40814_2017_211_MOESM1_ESM.docx]

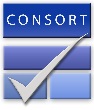
CONSORT 2010 checklist of information to include when reporting a randomised trial*

| Section/Topic | Item No | | | | Standard checklist item | | Extension for pilot trials | Reported on page No | | | |  |  |
| --- | --- | --- | --- | --- | --- | --- | --- | --- | --- | --- | --- | --- | --- |
| Title and abstract | | | |  | | |  |  | | | |  |  |
|  | 1a | | | | Identification as a randomised trial in the title | | Identification as a pilot or feasibility randomised trial in the title | 1 | | | |  |  |
|  | 1b | | | | Structured summary of trial design, methods, results, and conclusions (for specific guidance see CONSORT for abstracts) | | Structured summary of pilot trial design, methods, results, and conclusions (for specific guidance see CONSORT for abstracts) | 2 | | | |  |  |
| Introduction | |  | | | | |  |  | | | |  |  |
| Background and objectives | 2a | | | | Scientific background and explanation of rationale | | Scientific background and explanation of rationale for future definitive trial, and reasons for randomised pilot trial | 3 | | | |  |  |
|  | 2b | | | | Specific objectives or hypotheses | | Specific objectives or research questions for pilot trial | 3 | | | |  |  |
| Methods | | | |  | | |  |  | | | |  |  |
| Trial design | 3a | | | | Description of trial design (such as parallel, factorial) including allocation ratio | | Description of pilot trial design (such as parallel, factorial) including allocation ratio | 4 | | | |  |  |
|  | 3b | | | | Important changes to methods after trial commencement (such as eligibility criteria), with reasons | | Important changes to methods after pilot trial commencement (such as eligibility criteria), with reasons | 7 | | | |  |  |
| Participants | 4a | | | | Eligibility criteria for participants | |  | 4 | | | |  |  |
|  | 4b  4c | | | | Settings and locations where the data were collected | | How participants were identified and consented | 4  4-5, 17-18 | | | |  |  |
| Interventions | 5 | | | | The interventions for each group with sufficient details to allow replication, including how and when they were actually administered | |  | 5 | | | |  |  |
| Outcomes | 6a | | | | Completely defined pre-specified primary and secondary outcome measures, including how and when they were assessed | | Completely defined prespecifed assessments or measurements to address each pilot trial objective specified in 2b, including how and when they were assessed | 5-6 | | | |  |  |
|  | 6b  6c | | | | Any changes to trial outcomes after the trial commenced, with reasons | | Any changes to pilot trial assessments or measurements after the pilot trial commenced, with reasons  If applicable, prescpecified criteria used to judge whether, or how, to proceed with future definitive trial | 7-8  Table 1 | | | |  |  |
| Sample size | 7a | | | | How sample size was determined | | Rationale for numbers in the pilot trial | 7 | | | |  |  |
|  | 7b | | | | When applicable, explanation of any interim analyses and stopping guidelines | |  | N/A | | | |  |  |
| Randomisation: |  | | | |  | |  |  | | | |  |  |
| Sequence generation | 8a | | | | Method used to generate the random allocation sequence | |  | 5 | | | |  |  |
|  | 8b | | | | Type of randomisation; details of any restriction (such as blocking and block size) | | Type of randomisation (s); details of any restriction (such as blocking and block size) | 5 | | | |  |  |
| Allocation concealment mechanism | 9 | | | | Mechanism used to implement the random allocation sequence (such as sequentially numbered containers), describing any steps taken to conceal the sequence until interventions were assigned | |  | 5 | | | |  |  |
| Implementation | 10 | | | | Who generated the random allocation sequence, who enrolled participants, and who assigned participants to interventions | |  | 5 | | | |  |  |
| Blinding | 11a | | | | If done, who was blinded after assignment to interventions (for example, participants, care providers, those assessing outcomes) and how | |  | 5 | | | |  |  |
|  | 11b | | | | If relevant, description of the similarity of interventions | |  | N/A | | | |  |  |
| Statistical methods | 12a | | | | Statistical methods used to compare groups for primary and secondary outcomes | | Methods used to address each pilot trial objective whether qualitative or quantitative | Table 1 and page 7 | | | |  |  |
|  | 12b | | | | Methods for additional analyses, such as subgroup analyses and adjusted analyses | | Not applicable | N/A | | | |  |  |
| Results | | |  | | |  | |  | | |  | | |
| Participant flow (a diagram is strongly recommended) | 13a | | | | For each group, the numbers of participants who were randomly assigned, received intended treatment, and were analysed for the primary outcome | | For each group, the numbers of participants who were approached and/or assessed for eligibility, randomly assigned, received intended treatment, and were assessed for each objective | 8-9, Figure 1 | | | |  |  |
|  | 13b | | | | For each group, losses and exclusions after randomisation, together with reasons | |  | 8-9, Figure 1 | | | |  |  |
| Recruitment | 14a | | | | Dates defining the periods of recruitment and follow-up | |  | 8 | | | |  |  |
|  | 14b | | | | Why the trial ended or was stopped | | Why the pilot trial ended or was stopped | N/A | | | |  |  |
| Baseline data | 15 | | | | A table showing baseline demographic and clinical characteristics for each group | |  | Table 2 | | | |  |  |
| Numbers analysed | 16 | | | | For each group, number of participants (denominator) included in each analysis and whether the analysis was by original assigned groups | | For each objective, number of participants (denominator) included in each analysis. If relevant, these numbers should be by randomised group | 8-11 | | | |  |  |
| Outcomes and estimation | 17a | | | | For each primary and secondary outcome, results for each group, and the estimated effect size and its precision (such as 95% confidence interval) | | For each objective, results including expressions of uncertainty (such as 95% confidence interval) for any estimates. If relevant, these results should be by randomised group | 8-11 and Appendix 2, tables A1-3 | | | |  |  |
|  | 17b | | | | For binary outcomes, presentation of both absolute and relative effect sizes is recommended | | Not applicable | N/A | | | |  |  |
| Ancillary analyses | 18 | | | | Results of any other analyses performed, including subgroup analyses and adjusted analyses, distinguishing pre-specified from exploratory | | Results of any other analyses performed that could be used to inform the future definitive trial | N/A | | | |  |  |
| Harms | 19  19a | | | | All important harms or unintended effects in each group (for specific guidance see CONSORT for harms) | | If relevant, other important unintended consequences | N/A | | | |  |  |
| Discussion | | | | | | |  |  | | | |  |  |
| Limitations | 20 | | | | Trial limitations, addressing sources of potential bias, imprecision, and, if relevant, multiplicity of analyses | | Pilot trial limitations, addressing sources of potential bias and remaining uncertainty about feasibility | 12,16 | | | |  |  |
| Generalisability | 21 | | | | Generalisability (external validity, applicability) of the trial findings | | Generalisability (applicability) of pilot trial methods and findings to future definitive trial and other studies | 12-14 | | | |  |  |
| Interpretation | 22  22a | | | | Interpretation consistent with results, balancing benefits and harms, and considering other relevant evidence | | Interpretation consistent with pilot trial objectives and findings, balancing potential benefits and harms, and considering other relevant evidence  Implications for progression from pilot to future definitive trial, including any proposed amendments | 12-14, 17 | | | |  |  |
| Other information | | | | | | | |  |  |  | | |  |
| Registration | 23 | | | | Registration number and name of trial registry | | Registration number for pilot trial and name of trial registry | 4 | | | |  |  |
| Protocol | 24 | | | | Where the full trial protocol can be accessed, if available | | Where the pilot trial protocol can be accessed, if available | 4 | | | |  |  |
| Funding | 25 | | | | Sources of funding and other support (such as supply of drugs), role of funders | |  | 18 | | | |  |  |
|  | 26 | | | |  | | Ethical approval or approval by research review committee, confirmed with reference number | 17-18 | | | |  |  |

*We strongly recommend reading this statement in conjunction with the CONSORT 2010 Explanation and Elaboration for important clarifications on all the items. If relevant, we also recommend reading CONSORT extensions for cluster randomised trials, non-inferiority and equivalence trials, non-pharmacological treatments, herbal interventions, and pragmatic trials. Additional extensions are forthcoming: for those and for up to date references relevant to this checklist, see [www.consort-statement.org](http://www.consort-statement.org).
