# Supplementary material for: Fish oil supplementation in chronic obstructive pulmonary disease: feasibility of conducting a randomised controlled trial
Source: Pilot Feasibility Stud. 2017 Nov 25;3:66. doi: 10.1186/s40814-017-0211-2 (PMC5702222; doi:10.1186/s40814-017-0211-2)
Supplement: Supplementary file 2 — Table S1. Pulmonary function results including spirometry, lung volume, impulse oscillometry and gas transfer. Table S2. Results of well-being questionnaires including the hospital anxiety and depression scale and the chronic respiratory disease questionnaire. Table S3. Six-minute walk test and dyspnoea results. (DOCX 21 kb) [file 40814_2017_211_MOESM2_ESM.docx]

Appendix 2

*Pulmonary function*

**Table** A1 Pulmonary function results including spirometry, lung volume, impulse oscillometry and gas transfer.

|  | **Fish oil (n=3)** | | **Placebo (n=5)** | |  |
| --- | --- | --- | --- | --- | --- |
|  | **Baseline**  **Median (IQR)** | **Post**  **Median (IQR)** | **Baseline**  **Median (IQR)** | **Post**  **Median (IQR)** | **Effect size (95% CI)** |
| **Spirometry^a^** |  |  |  |  |  |
| FEV_1_ (L) | 1.62 (0.67) | 1.49 (0.70) | 1.45 (0.72) | 1.44 (0.67) | -0.21 (-0.28, -0.14) |
| FVC (L) | 2.93 (0.91) | 2.74 (0.76) | 3.12 (0.83) | 3.43 (0.72) | -1.24 (-1.35, -1.12) |
| FEV_1_/FVC | 0.55 (0.04) | 0.55 (0.07) | 0.46 (0.06) | 0.47 (0.03) | 0.41 (0.39, 0.43) |
| FEV_1_ % predicted | 70.00 (10.00) | 68.00 (3.50) | 54.00 (23.00) | 54.00 (28.00) | -1.07 (-5.02, 2.89) |
| FEF_25-75_ | 0.57 (0.32) | 0.51 (0.52) | 0.49 (0.24) | 0.43 (0.28) | 0.91 (0.55, 1.28) |
| **Lung volume^b^** |  |  |  |  |  |
| IC (L) | 2.50 (0.50) | 2.61 (0.71) | 2.50 (0.72) | 2.33 (0.43) | 0.63 (0.35, 0.90) |
| V_T_ (L) | 0.92 (0.30) | 0.83 (0.11) | 0.97 (0.15) | 1.02 (0.18) | -0.61 (-0.74, -0.48) |
| FRC (L) | 3.95 (0.05) | 3.68 (0.10) | 4.26 (1.15) | 4.43 (1.11) | -1.99 (-2.20, -1.78) |
| RV (L) | 3.02 (0.02) | 2.74 (0.18) | 3.51 (0.94) | 3.30 (0.50) | -0.99 (-1.18, -0.79) |
| **Impulse oscillometry^a^** |  |  |  |  |  |
| R5 (kpa/L/s) | 0.44 (0.04) | 0.39 (0.01) | 0.43 (0.10) | 0.46 (0.17) | -1.03 (-1.05, -1.01) |
| R20 (kpa/L/s) | 0.32 (0.08) | 0.33 (0.04) | 0.29 (0.01) | 0.30 (0.08) | 0.29 (0.27, 0.31) |
| R5-R20 (kpa/L/s) | 0.12 (0.03) | 0.09 (0.04) | 0.07 (0.24) | 0.15 (0.07) | -1.31 (-1.38, -1.24) |
| X5 (kpa/L/s) | -0.18 (0.01) | -0.14 (0.09) | -0.18 (0.14) | -0.20 (0.13) | -0.92 (-0.96, -0.88) |
| **Gas Transfer^b^** |  |  |  |  |  |
| DLco (ml STPD.min-1.mm HG-1) | 13.32 (5.25) | 9.34 (6.25) | 16.43 (10.22) | 17.54 (9.96) | -1.04 (-1.46, -0.63) |
| DLco% predicted | 53.50 (16.50) | 47.00 (21.00) | 67.00 (39.00) | 71.00 (40.00) | -0.89 (-3.30, 1.51) |
| VA (L) | 4.40 (0.68) | 3.94 (0.71) | 5.35 (0.42) | 5.45 (0.39) | -1.41 (-1.47, -1.34) |
| DLco/VA | 2.91 (0.75) | 2.73 (0.95) | 3.21 (1.06) | 3.23 (0.78) | -0.22 (-0.36, -0.09) |

^a^ Post bronchodilator; ^b^Pre bronchodilator

CI, confidence interval; DLco, diffusing capacity of the lung to carbon monoxide; FEV_1_, forced expiratory volume in 1 second; FEV1/FVC, forced expiratory volume in 1 second/forced vital capacity; FEV_1_ % predicted, forced expiratory volume in 1 second percent of predicted; FEF_25-75_, forced expiratory flow 25-75% of maximum; FRC, functional residual capacity; FVC, forced vital capacity; IC, inspiratory capacity; IQR, interquartile range; L, Litre; ml, millilitre; mmHG, millimetres of mercury; R5, resistance at 5hz; R20, resistance at 20hz; RV, residual volume; STPD, standard temperature pressure dry; V_T_, tidal volume; VA, lung capacity; X5, reactance at 5 Htz.

*Well-being questionnaires*

**Table A2** Results of well-being questionnaires including the hospital anxiety and depression scale and the chronic respiratory disease questionnaire.

|  | **Fish oil (n=3)** | | **Placebo (n= 5)** | |  |
| --- | --- | --- | --- | --- | --- |
|  | **Baseline**  **Median (IQR)** | **Post**  **Median (IQR)** | **Baseline**  **Median (IQR)** | **Post**  **Median (IQR)** | **Effect size (95% CI)** |
| **HADS** |  |  |  |  |  |
| Anxiety | 7.00 (5.50) | 5.0 (1.00) | 6.00 (1.00) | 4.0 (3.00) | -0.48 (-2.51, 1.56) |
| Depression | 5.00 (3.00) | 1.00 (1.50) | 4.00 (1.00) | 3.0 (2.00) | -1.33 (-2.83, 0.17) |
| **CRQ** |  |  |  |  |  |
| Total score | 4.75 (0.20) | 5.00 (0.25) | 5.40 (1.30) | 5.50 (0.98) | -0.34 (-0.54, -0.13) |
| Dyspnoea | 5.40 (1.30) | 6.20 (1.40) | 4.80 (3.00) | 4.80 (2.00) | -0.79 (-1.15, -0.43) |
| Fatigue | 4.75 (0.38) | 5.00 (0.63) | 4.50 (1.00) | 4.25 (0.75) | 0.60 (0.23, 0.98) |
| Emotional function | 3.71 (0.64) | 5.00 (0.79) | 5.71 (1.14) | 6.43 (1.00) | 0.50 (-0.33, 0.44) |
| Mastery | 5.00 (1.50) | 5.25 (0.88) | 6.50 (2.50) | 6.25 (1.25) | -0.70 (-1.15, -0.26) |

CI, confidence interval; CRQ, chronic respiratory questionnaire; HADS, hospital anxiety and depression scale; IQR, interquartile range

*Functional exercise capacity and dyspnoea*

**Table A3** Six minute walk test and dyspnoea results

|  | **Fish oil (n=3)** | | **Placebo (n=5)** | |  |
| --- | --- | --- | --- | --- | --- |
|  | **Baseline**  **Median (IQR)** | **Post**  **Median (IQR)** | **Baseline**  **Median (IQR)** | **Post**  **Median (IQR)** | **Effect size (95% CI)** |
| **Walk distance (m)** | 529 (46) | 519.5 (51.5) | 405 (128) | 452 (95) | -0.50 (-23.56, 22.57) |
| **VAS intensity (mm)*** | 22.13 (19.88) | 31.88 (14.38) | 12.00 (9.10) | 12.00 (17.25) | 0.21 (-14.46, 14.89) |
| **VAS unpleasantness (mm)*** | 8.13 (3.88) | 23.13 (11.38) | 6.00 (18.00) | 3.00 (6.75) | 0.58 (-23.95, 25.10) |
| **Dyspnoea-12*** |  |  |  |  |  |
| Total | 7.00 (1.00) | 6.00 (1.00) | 12.00 (16.00) | 4.00 (5.00) | 1.05 (-3.61, 5.71) |
| Physical | 6.00 (1.00) | 5.5 (1.50) | 6.00 (8.00) | 2.00 (3.00) | 1.07 (-1.35, 3.49) |
| Affective | 1.00 (0.00) | 0.5 (0.5) | 6.00 (8.00) | 0.00 (2.00) | 0.90 (-1.67, 3.46) |

*These tests were performed pre and post six minute walk; data are reported for pre walk assessment only

IQR, interquartile range; m, metre; mm, millimetre; VAS, visual analogue scale.
